# The Evolution and Development of Cephalopod Chambers and Their Shape

**DOI:** 10.1371/journal.pone.0151404

**Published:** 2016-03-10

**Authors:** Robert Lemanis, Dieter Korn, Stefan Zachow, Erik Rybacki, René Hoffmann

**Affiliations:** 1 Institute of Geology, Mineralogy, and Geophysics, Ruhr-Universität Bochum, Bochum, Germany; 2 Museum für Naturkunde Berlin, Leibniz-Institut für Evolutions- und Biodiversitätsforschung, Berlin, Germany; 3 Department of Scientific Visualization and Data Analysis, Zuse Institute, Berlin, Germany; 4 Helmholtz-Zentrum Potsdam, Deutsches GeoForschungsZentrum, Potsdam, Germany; University of California, UNITED STATES

## Abstract

The Ammonoidea is a group of extinct cephalopods ideal to study evolution through deep time. The evolution of the planispiral shell and complexly folded septa in ammonoids has been thought to have increased the functional surface area of the chambers permitting enhanced metabolic functions such as: chamber emptying, rate of mineralization and increased growth rates throughout ontogeny. Using nano-computed tomography and synchrotron radiation based micro-computed tomography, we present the first study of ontogenetic changes in surface area to volume ratios in the phragmocone chambers of several phylogenetically distant ammonoids and extant cephalopods. Contrary to the initial hypothesis, ammonoids do not possess a persistently high relative chamber surface area. Instead, the functional surface area of the chambers is higher in earliest ontogeny when compared to *Spirula spirula*. The higher the functional surface area the quicker the potential emptying rate of the chamber; quicker chamber emptying rates would theoretically permit faster growth. This is supported by the persistently higher siphuncular surface area to chamber volume ratio we collected for the ammonite *Amauroceras* sp. compared to either *S*. *spirula* or nautilids. We demonstrate that the curvature of the surface of the chamber increases with greater septal complexity increasing the potential refilling rates. We further show a unique relationship between ammonoid chamber shape and size that does not exist in *S*. *spirula* or nautilids. This view of chamber function also has implications for the evolution of the internal shell of coleoids, relating this event to the decoupling of soft-body growth and shell growth.

## Introduction

**Fig 1:**
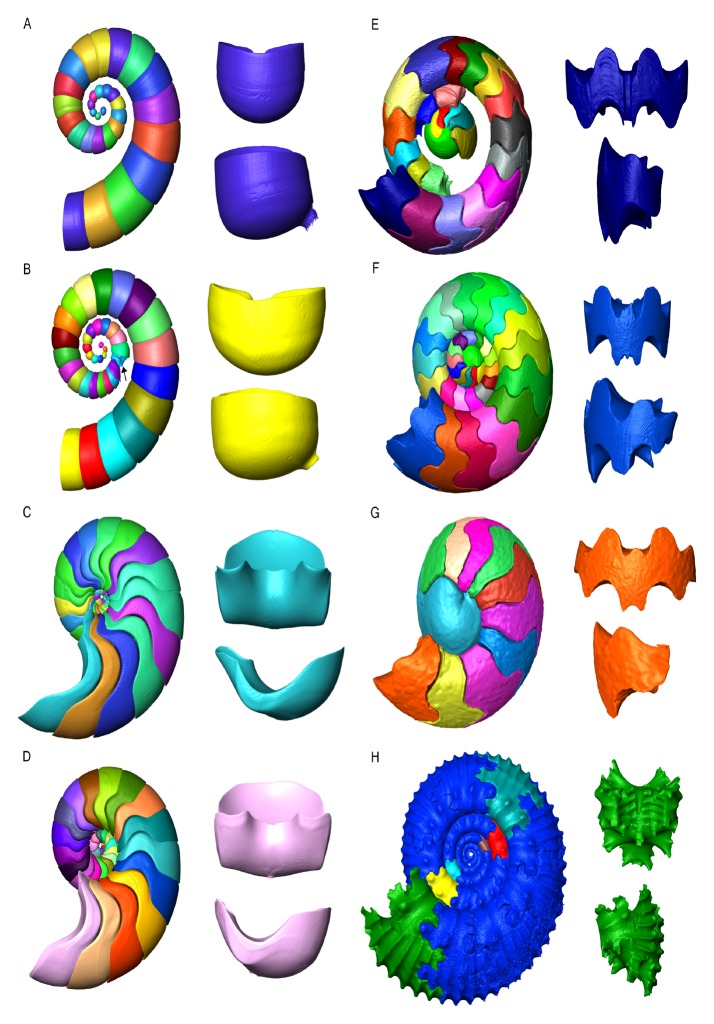
Three-dimensional surface renderings of the segmented chambers of all specimens used in this study. A) *Spirula spirula*, B) pathological *S*. *spirula* (pathological chamber indicated by black arrow), C) *Nautilus pompilius* D) *Allonautilus scrobiculatus* E) *Arnsbergites* sp. F) *Amauroceras* sp. G) *Cadoceras* sp. H) *Kosmoceras* sp. Segmented chambers appear in sequentially different colors; only six chambers of *Kosmoceras* were segmented. The largest segmented chamber is shown in dorsal/ventral view (top) and lateral view (bottom). The boundaries of the chamber volumes trace the shape of the septa. Images are not to scale.

Cephalopods are a group of marine mollusks that evolved in the Cambrian from a monoplacophoran-like ancestor [1–4]; the earliest known cephalopod is the Late Cambrian *Plectronoceras* [5,6]. Basal cephalopods possess a phragmocone that is distinct from other mollusk shells (conch) by the division into discrete chambers (Fig 1). The chambers are separated by mineralized partitions called septa that allows the shell to function as a buoyancy device. The multi-chambered, aragonitic cephalopod shell is a key adaptation that allows the animal to dwell in the water column without constantly expending energy [7,8]. A thin organic strand, called the siphuncle, runs through all phragmocone chambers and connects this with the rear of the soft body that sits in the body chamber. Liquid and gas diffuse into and out of the chambers through the siphuncle and thereby allows for buoyancy adjustments [9,10]. The siphuncle was supported by the connecting pellicle, a thin (sub-micron) proteinaceous structure composed of conchiolin, that covers the inner surface of each chamber which stores and transports liquid to the siphuncle [9].

The evolution of the chambered cephalopod shell allowed for a life-habit in the water column facilitated by neutral buoyancy; this led to the diversification of the cephalopods that can be classified into three major groups: “Nautiloidea”, Ammonoidea, and Coleoidea [3,11]. Nautiloidea are represented by the two extant genera: *Allonautilus* and *Nautilus*, referred to collectively as nautilids. Ammonoidea, which originated from the Bactritida about 417 million years ago during the Early Devonian and went extinct at the end of the Cretaceous [12], have been one of the prime groups to study evolutionary biology through geologic time [13–16].

Nautilids and ammonoids possess external shells. However, the coleoids that also originated from Bactritida, evolved an internal shell that was highly reduced or completely lost in the majority of derived taxa [3]. Recently, the ammonoids and the bactritids have been considered as stem group coleoids [3,6]. Coleoids comprise the majority of Recent cephalopod diversity; only the sepiids and the deep sea squid *Spirula spirula* have retained a mineralized phragmocone. *S*. *spirula* is the only extant coleoid with a fully developed spiral phragmocone [17]. The shells of Nautiloidea, Ammonoidea, and Coleoidea show characteristic differences both in their overt morphology and in the morphology of their septa. The septa of *S*. *spirula* are semi-hemispherical structures (Fig 1A and 1B) similar to the septa of most of the nautiloids (Fig 1C and 1D). However, the fossil record is replete with more complicated septal structures in nautiloids that show variable degrees of foldings turning their septa into multilobate structures [7,18].

Septal complexity reached its apex in the ammonoids (Fig 1E), more specifically in the Jurassic and Cretaceous ammonites that show highly complex folded septa (Fig 1F–1H). Ammonoids show a persistent, iterative evolutionary trend towards increasing septal complexity [19,20]. The most common explanation of this evolutionary trend is either mechanical [21] or physiological [22]. This paper focuses on potential physiological drivers for the morphological evolution of cephalopod phragmocone chambers. The morphology of these chambers are influenced by three variables: shell wall morphology, septal morphology, and septal spacing. While tomographic data does present an opportunity to test mechanical hypothesis, these are beyond the scope of this work and will be the focus of future research.

Ammonoids have been argued to possess a relatively large chamber surface area due to shell morphology—such as whorl overlap—and, more commonly, septal morphology [22–25]. The increased folding of the septa is thought to increase the surface area of the chambers [26] leading to a range of physiological hypotheses for septa such as those of Kröger [22] who argued septal complexity increases the relative surface area and volume of the pellicle [9,10,27,28], which allows a greater degree of buoyancy compensation due to retention of a greater volume of liquid. Increasing the relative surface area of a membrane, in our case this is the pellicle and siphuncular epithelium, is a common adaptation in biology that allows a maximization of fluid transport [29]. Increased rates of buoyancy change and enhanced respiration are further hypotheses implementing physiological functions to explain septal complexity [13,24,30–32]. These hypotheses tend to depend on a relatively high functional surface area, here defined as the ratio between surface area and volume. The previous studies [9,13,22,24,31,32] lead to the first hypothesis tested herein: the surface area to volume ratio of the phragmocone chambers will be higher in ammonoids than in either *S*. *spirula* or the nautilids. Growth is partially dependent on emptying rates as the chamber formation cycle is connected to the emptying of the prior chamber and the buoyancy of the animal depends on fluid being removed from the chambers as shell material is added [33,34]. A second hypothesis focuses on the siphuncle, because some authors [35] argue that it is the siphuncle, not the pellicle, which is the major constraint of diffusion into and out of the chambers. If ammonites increased the functional area of the siphuncle relative to chamber volume compared to nautilids, as suggested by Ward [36], then the surface area of the siphuncle vs. chamber volume should be higher in ammonoids than in nautilids and *S*. *spirula*.

Volume and surface area are notoriously difficult to measure directly in fossil shells hence the classical dependence on volumetric reconstructions based on simple geometric shapes [37–40]. These reconstructions are ultimately incapable of fully describing biological structures and accounting for changes in growth through ontogeny. This has led to the application of tomographic techniques, computed tomography [41–43] and grinding tomography [44,45], in order to directly quantify shell and chamber volume. Tomographic data are available for chamber volumes but not for surface area. Therefore, we reconstruct the surface area and volume trajectories of: *A*. *scrobiculatus*, *N*. *pompilius*, *Cadoceras* sp., *Kosmoceras* sp., *Amauroceras* sp., *Arnsbergites* sp., and *S*. *spirula*.

Surface area scales with the square of length while volume scales with the cube of length; maintenance of a constant surface area to volume ratio through ontogeny requires changes in shape to compensate for the disproportionate scaling between surface area and volume [46]. As an object increases in size alone, the ratio of surface area to volume will decrease regularly. We therefore expect an overall decreasing trend in the surface area to volume ratios as the chambers increase in all specimens. The comparisons we will focus on in this paper are twofold: firstly, the surface area to volume ratios between specimens at equivalent sizes and, secondly, the Vogel number. The Vogel number is the ratio of the linearized surface area and volume which eliminates the effects of scaling differences between surface area and volume. i.e., if a shape increases its dimensions in constant proportion the Vogel Number will be a constant value even through the SA:V will decrease. If the ratios of interest are only influenced by size then all specimens should show the same ratio at a specific size.

We present the first study of the ontogenetic and evolutionary change in the surface area and volume ratios of cephalopod shell chambers (SA_C_:V_C_) and ratios of siphuncular surface area and chamber volume (SA_S_:V_C_).

Specifically, we test the following hypotheses derived earlier:

1. Ammonoids will have a consistently higher SA_C_:V_C_ ratio than, nautilids, and *S*. *spirula*.
2. Ammonoids will also possess a higher SA_S_:V_C_ than the nautilids.

## Material and Methods

All fossil specimens used in this study are stored in the Ruhr-Universität Bochum (RUB), Universitätsstrasse 150, Bochum 44801, Germany and are accessible to interested parties. Specimen designations: *Cadoceras* sp.: RUB-Pal 11245, *Kosmoceras* sp.: RUB-Pal 11246, *Allonautilus scrobiculatus*: RUB-Pal 11247, *Nautilus pompilius*: RUB-Pal 11248, *Spirula spirula*: RUB-Pal 11249, *Spirula spirula* (pathological): RUB-Pal 11250, *Amauroceras* sp.: RUB-Pal 11251, *Arnsbergites* sp.: MB.C.25122. No permits were required for the described study, which thus complied with all relevant regulations.

### Specimens

A total of eight shells were used in this study (Table 1). Computed tomographic scans were produced for all specimens and the data were processed with ZIBAmira (Zuse Institute, Berlin-ZIB). Processing tomographic data is very time intensive and computed tomography (CT) requires hollow fossil preservation in order to have the highest precision. Due to this, the number of specimens available for this study is limited due to the extreme rarity of this type of material; however, we used specimens from several geological periods from the Palaeozoic and Mesozoic and specimens with a variety of morphologies. The genera *Allonautilus*, *Nautilus*, and *Spirula* represent the only extant forms that possess a fully formed, spiral phragmocone. Fossil cephalopods with four different morphologies, reflected by differences in chamber geometry, are presented in this study. Three of them are represented by Jurassic ammonites: *Amauroceras* sp. (Pliensbachian), *Cadoceras* sp. (Callovian) and *Kosmoceras* sp. (Callovian). The fourth morphology is represented by the Carboniferous goniatite *Arnsbergites* sp. (Viséan).

**Table 1:** Specimen Data and Tomographic Scan Meta-Data.

Specimen	Age	Diameter (mm)	Tube:voltage (kV)/current (μA)	Data set dimensions	Voxel size (mm)	Average percent error of volume (+/-)
*Nautilus pompilius*	Recent	170	180/150	962x1008x560	0.17500	7.06746
*Allonautilus scrobiculatus*	Recent	177.13	100/350	2383x1746x3046	0.06000	2.31144
*S*. *spirula*	Recent	16.51	75/200	1880x1880x2200	0.00875	1.86725
*S*. *spirula* (path.)	Recent	19.09	150/150	894x774x338	0.02523	5.56178
*Cadoceras* sp.	Middle Jurassic	0.98	27.2	2048x2048x1948	0.00074	1.59767
*Amauroceras* sp.	Lower Jurassic	2.99	80/200	1432x2314x1829	0.00315	4.08041
*Arnsbergites* sp.	Mississippian Carboniferous	1.64	50/190	1880x1880x1700	0.00250	5.00604
*Kosmoceras* sp.	Upper Jurassic	13.06	120/350	2489x1677x2266	0.00815	1.73740

### CT Scanning

Micro-computed tomographic scans of *Nautilus pompilius* and the pathological specimen of *Spirula spirula* were performed at the Steinmann Institute at the University Bonn using a phoenix|x-ray|v|tome|x s (General Electric). Nanofocus-computed tomographic (nano-CT) scans of *Spirula spirula* and *Arnsbergites* sp. were done at the GeoForschungsZentrum (GFZ, Potsdam) using a phoenix nanotom-s (General Electric). *Allonautilus scrobiculatus*, *Kosmoceras* sp. and *Amauroceras* sp. were scanned using nano-CT at the TPW Prüfzentrum (Neuss, Germany) with a phoenix nanotom m (General Electric). *Cadoceras* sp. was scanned at the Advanced Photon Source at Argonne National Laboratory using phase contrast synchrotron radiation based micro-computed tomography. All data are deposited at the Ruhr Universität-Bochum. All recent specimens are adults as indicated by the presence of their terminal countdown morphology [47]; *Cadoceras* sp. is a juvenile while the other ammonoids are juvenile-sub-adult.

CT data were imported into ZIBAmira where the relevant volumes were segmented using the threshold function and manual selection function. All recent shells were completely segmented (i.e., shell and chamber volumes) while the ammonoid shell chambers were segmented where available as some chambers were damaged or not preserved (Fig 1C). The chambers of *Kosmoceras* sp. were segmented two per whorl for the final two and a half whorls (Fig 1G). The shells of *S*. *spirula*, *A*. *scrobiculatus* and *Amauroceras* sp. were segmented with the preserved siphuncular tube. Three chambers of the *Amauroceras* sp. specimen and the rest of the specimens did not preserve the siphuncle; however, the volume and surface area of the siphuncle was reconstructed from linear measurements, as described for *Cadoceras* sp. in [38], and subtracted from the volumes and surface areas for their respective chambers in each specimen. Comparisons between siphuncular area and chamber volume were performed on the three specimens that preserved the siphuncular tube: *A*. *scrobiculatus*, *S*. *spirula*, and *Amauroceras* sp.

All volume data was converted into surface files from which surface area and volume values were taken (S1 Table). Partial volume effects (PVE) are the primary source of error in the reconstruction of volumes [42]. In order to estimate the susceptibility of our data to such errors we transformed volume data for each specimen by expanding/shrinking it by one voxel layer relative to the total 3D volume. Percent error was calculated for each transform and the average error is presented in Table 1. This value is a representation of the variability of the data due to resolution and the segmentation process.

## Results

### Surface Area/Volume Ratios

**Fig 2:**
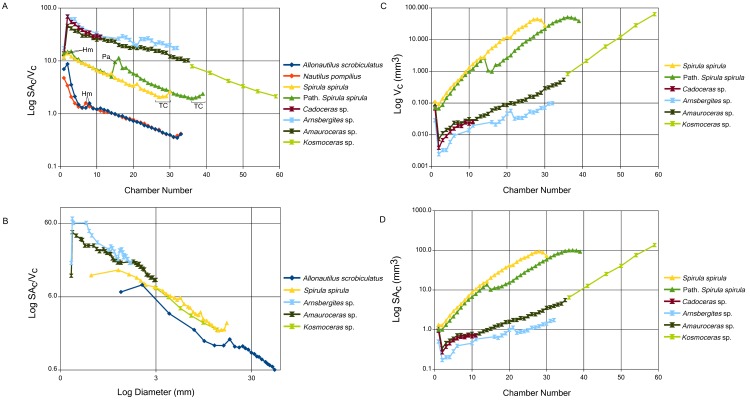
A) Comparison between the surface area to volume ratio (SA_C_:V_C_) of each segmented chamber against chamber number for all specimens. B) SA_C_:V_C_ against shell diameter at each chamber for *A*. *scrobiculatus*, *S*. *spirula*, *Arnsbergites* sp., *Amauroceras* sp., and *Kosmoceras* sp. SA_C_:V_C_ is a parameter that reflects the capacity of the shell to compensate for potential buoyancy changes due to the water storing, organic lining in each chamber (Kroger, 2002). Chamber volume (C) and chamber surface area (D) comparisons between *S*. *spirula* and selected ammonoids. *A*. *scrobiculatus* and *N*. *pompilius* have an overall larger volume and surface area due to the much larger size of the animal, maximum diameter is an order of magnitude larger than *S*. *spirula* or *Kosmoceras*. Comparison between *S*. *spirula* and the ammonoids is a comparison between extreme morphologies as *S*. *spirula* has a whorl interspace, conservative shell cross-section through ontogeny and simple sutures while ammonoids have overlapping whorls, more complex septa (complexity changes through ontogeny), and variable conch morphology and ornamentation. Hm is the potential hatching point, Pa is the pathological chamber, TC is the terminal countdown.

Comparisons of chamber surface area/ chamber volume (SA_C_:V_C_) against chamber number are shown in Fig 2A. In general, closely related taxa show similar SA_C_:V_C_ ratios for each equivalent chamber; nautilids plot together as do the *S*. *spirula* and ammonoid shells. All tested shells show a decreasing SA_C_:V_C_ through ontogeny as expected from simple scaling rules. Interspecific comparisons are done on the basis of shell diameter (Fig 2B). Nautilids show the lowest SA_C_:V_C_ relative to diameter, with a maximum ratio of 8.75 in the 2^nd^ chamber of *A*. *scrobiculatus* and a minimum ratio of 0.35 in the 32^nd^ chamber of *A*. *scrobiculatus*. Ammonoids possess the largest ratios for diameters under 3 mm (Fig 2B) with a maximum ratio of 70.38 in the second chamber of *Arnsbergites* sp. and a minimum ratio of 2.14 in the 59^th^ chamber of *Kosmoceras* sp. All ammonoids, regardless of stratigraphic age, show similar SA_C_:V_C_ ratios throughout ontogeny. The SA_C_:V_C_ ratios of ammonoids attain the highest overall values, however, they reach the same values as *S*. *spirula* in later ontogeny.

Nautilids and *S*. *spirula* shells show slight upturns in the SA_C_:V_C_ ratios in the last chambers reflecting their terminal countdown with septal crowding [47,48]. The pathological *S*. *spirula* shell shows a sudden increase in its SA_C_:V_C_ corresponding to a non-lethal injury of its phragmocone (reported for the first time)—visible as the dorso-ventral compaction of one chamber—that results in a permanent offset from the non-pathological shell that lasts through ontogeny. SA_C_:V_C_ in chamber 15 in the non-pathological shell is 4.79 while the same chamber in the pathological shell has a ratio of 9.52. The initial chamber of ammonoids and *S*. *spirula* show similar values, both have a spherical to ellipsoidal shape and similar diameters. Ammonoids show a much higher increase in SA_C_:V_C_ between the initial chamber and the subsequent chamber (303.98% in *Arnsbergites* sp. and 293.89% in *Amauroceras* sp.) than *S*. *spirula* (17.83% in the non-pathological shell and 5.97% in the pathological shell) due to the large decrease in size in the second chamber compared to the initial chamber. Comparison of chamber volume (Fig 2C) and chamber surface area (Fig 2D) between *S*. *spirula* and the ammonoid specimens shows that *S*. *spirula* possess a persistently higher relative chamber volume through most of ontogeny, corresponding to a higher surface area as well, though *Kosmoceras* sp. attains a slightly higher surface area and volume by the last segmented chamber despite *Kosmoceras* sp. and *S*. *spirula* having similar final shell diameters (Table 1).

### Siphuncular Surface vs. Chamber Volume

**Fig 3:**
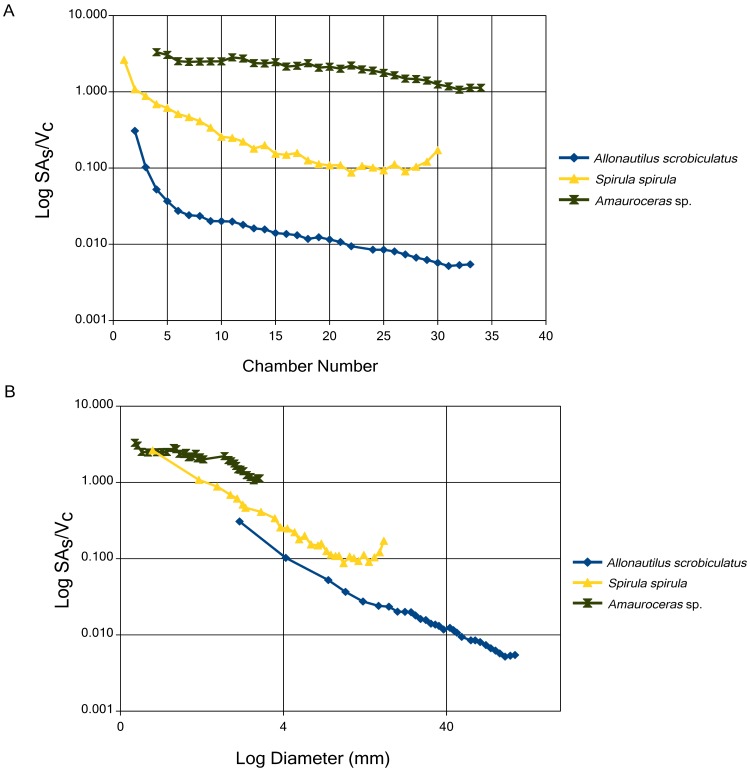
Siphuncular surface area to chamber volume ratio (SA_S_:V_C_) for the three specimens that preserve the siphuncular tube: *A*. *scrobiculatus*, *S*. *spirula*, and *Amauroceras*, plotted against chamber number (A) and shell diameter (B). The siphuncle transfers liquid and gas into and out of the shell, therefore the surface area of the siphuncle limits the diffusion rates of liquid/gas. The higher the SA_S_:V_C_ the higher the potential rate of diffusion.

SA_S_:V_C_ trends are unsurprisingly similar to SA_C_:V_C_ trends in that both show a general decrease through ontogeny and similar differentiation between taxa (Fig 3A). *Amauroceras* sp. shows the highest values (maximum of 3.30), while the specimen of *A*. *scrobiculatus* shows the lowest values (minimum of 0.005), and that of *S*. *spirula* lying between them with the overlap with *Amauroceras* sp. being the initial chamber (Fig 3B). SA_S_:V_C_ values show a lower rate of decrease through ontogeny, slight increases in the trend are seen in the terminal chambers of *S*. *spirula* and *A*. *scrobiculatus* (Fig 3).

Comparison between a hypothetical, reconstructed siphuncle volume and surface area and true volume and surface area were made with the data from *Amauroceras* sp. The siphuncle was reconstructed as a series of connected cylinders spanning the length of each chamber. Measurements for the cylinder were taken along the siphuncular foramen—the region of the septum through which the siphuncle passes through—see [41]. Maximum percent error for siphuncle volume was 60%, average percent error was 25%. Maximum error for siphuncle surface area was 25%, average error was 10%. Hypothetical siphuncle measurements were used in specimens that have not preserved the siphuncle to correct the chamber volume and surface area. Despite the significant error this was done because the relative contribution of the siphuncle to the total volume/surface area is minor even in small chambers. However, due to the potentially high error, SA_S_:V_C_ were confined to specimens that preserved the siphuncle and no comparisons were made between the preserved siphuncles and the reconstructed siphuncles.

## Discussion

### Scaling

**Fig 4:**
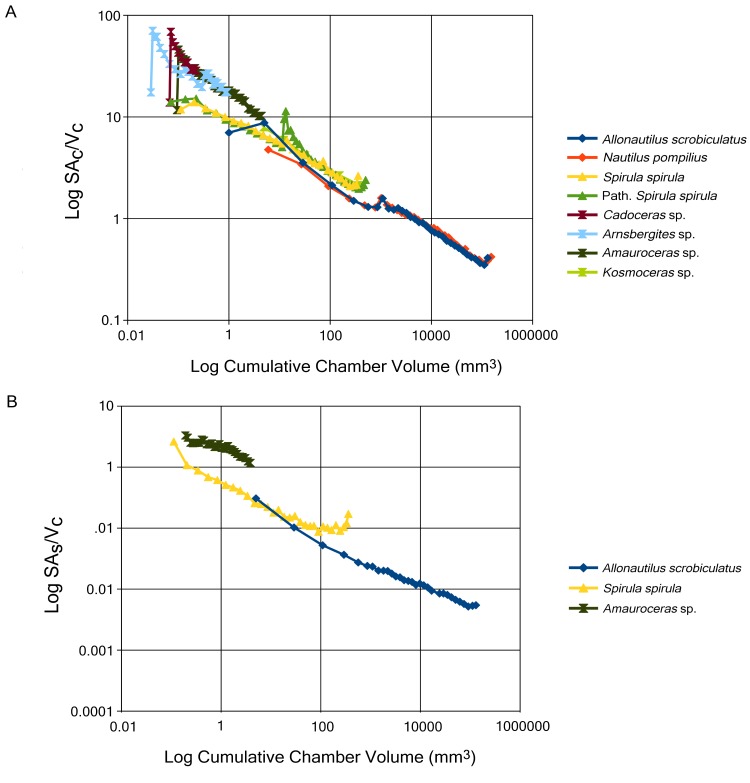
Chamber surface area to chamber volume ratio (A) and siphuncular surface area to chamber volume ratio (B) plotted against cumulative chamber volume. While both shell diameter and cumulative volume are proxies for size, volume is a more accurate basis for comparison due to the heteromorphic morphology of the shell of *Spirula*, possessing a whorl interspace that artificially inflates shell diameter. Regardless both graphs show that ammonoids possess a relatively high surface area to volume ratio in early ontogeny.

A short discussion of scaling is necessary in order to properly contextualize our data. It is well known that surface area to volume ratios inversely scale with size, the larger the object the lower the SA:V compared to a smaller, equivalent shape [46]. The clear differentiation of groups when ratios and values are plotted against chamber number (Figs 2A, 2C, 2D and 3A) is an effect of scaling. These graphs are not a reliable basis for interspecific comparisons but do illustrate the trends for each group. In order to account for size scaling, comparisons are done against shell diameter, Figs 2B and 3B. If size was the only factor affecting SA:V values then we would expect to see the same SA:V in each taxon for a given shell diameter. There is a complication in this regard since *S*. *spirula* possesses a whorl interspace that artificially inflates the shell diameter. In order to correct for this, the SA:V ratios are plotted against cumulative chamber volume (Fig 4). Comparison between shell diameter and cumulative chamber volumes shows the same trends and further illustrates the difference between SA_C_:V_C_ and SA_S_:V_C_ in early ammonoid ontogeny. While the difference in ratios between *S*. *spirula* and the nautilids decreases when compared to cumulative volume, it does not disappear completely demonstrating the influence of shape on these ratios.

**Fig 5:**
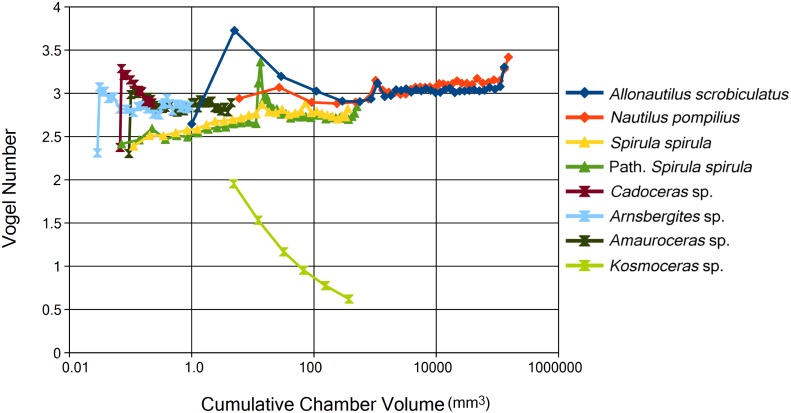
Calculated Vogel number for each specimen used in this study. Vogel number is calculated as the square root of the surface area of the chamber divided by the cube root of the volume of the chamber. Linearizing these values allow direct comparisons between the two while removing scaling effects due to size. It is important to note that the difference between ammonites and *S*. *spirula* in early ontogeny exists even when corrected for size. The high values shown by the early chambers of *A*. *scrobiculatus* may be an artifact due to resolution and should be interpreted with care.

Additionally, surface area and volume can be linearized by taking the square root of surface area and the cube root of volume to calculate the Vogel number. The Vogel number is a shape parameter that is independent of size, yet still shows the same trends in early ontogeny between ammonoids and *S*. *spirula* (Fig 5). *Kosmoceras* sp. shows a very different trend in Vogel number compared to all other specimens, namely it demonstrates a strong dependence of shape on size. As Vogel number is, in a way, an index of flatness it might be expected to be strongly dependent on septal angle; however, this seems not to be the case (S1 Fig). Indeed the septal angle of the Jurassic ammonites and Carboniferous *Arnsbergites* sp. are quite different but all show similar SA_C_:V_C_ and Vogel numbers. Septal angles of the tested ammonoids and *S*. *spirula* converge in early ontogeny but this does not correlate to a convergence of SA_C_:V_C_ or Vogel number. The shape of the chamber is dependent on three morphological parameters: conch and septal morphology and septal angle. Disentangling the contribution of each of these parameters on overall conch shape is a complex topic and may be an invigorating avenue of future research. However, we anticipate that no one single parameter will completely describe shape differences.

### Siphuncle and SA_S_:V_C_

The expected recovery of a higher SA_S_:V_C_ in ammonoids is supported by our data (Fig 3B). The increase in the functional surface area of the siphuncle would increase the hypothetical limit of liquid and gas diffusion rate into and out of the chambers as the siphuncular soft tissue of ammonoids is not known to be very different than extant forms [49]. It has been observed that the connecting rings of ammonoids shows morphological features that suggest higher diffusion rates of liquid through the siphuncular epithelium [50,51,26]. Unfortunately it is impossible to take these morphological differences into consideration because there are no quantitative studies measuring fluid flow within the siphuncle and pellicle of *Nautilus* and *Spirula*. However, if ammonoids did indeed have a faster transmission of fluid into the siphuncle this would serve to further amplify the effect of the higher SA_S_:V_C_ we observe.

There are several ways to increase the functional siphuncle surface area: a) increasing the length of the siphuncle, b) increasing the cross-sectional diameter, and c) increase the folding of the siphuncular epithelium [52,53]. Increasing the linear length of the siphuncle would necessitate increasing septal spacing, thereby increasing the chamber volume at a faster rate than the siphuncle length per unit increase in septal spacing; a counter-productive result. Increasing the cross-sectional diameter of the siphuncular tube should decrease the mechanical strength of the tube making it more susceptible to breakage [54]. Ammonoids do however show a migration of the siphuncle to the ventral edge of the chamber. This migration maximizes the arc length of the siphuncle relative to a median position, such as that in the nautilids, or a dorsal position such as that in *S*. *spirula*. Maximizing the surface area of the siphuncle would permit higher potential growth rates. The faster growth of the chamber volume (i.e., cameral fluid) compared to siphuncular area would lead to decreasing growth rates through ontogeny.

### Curvature

**Fig 6:**
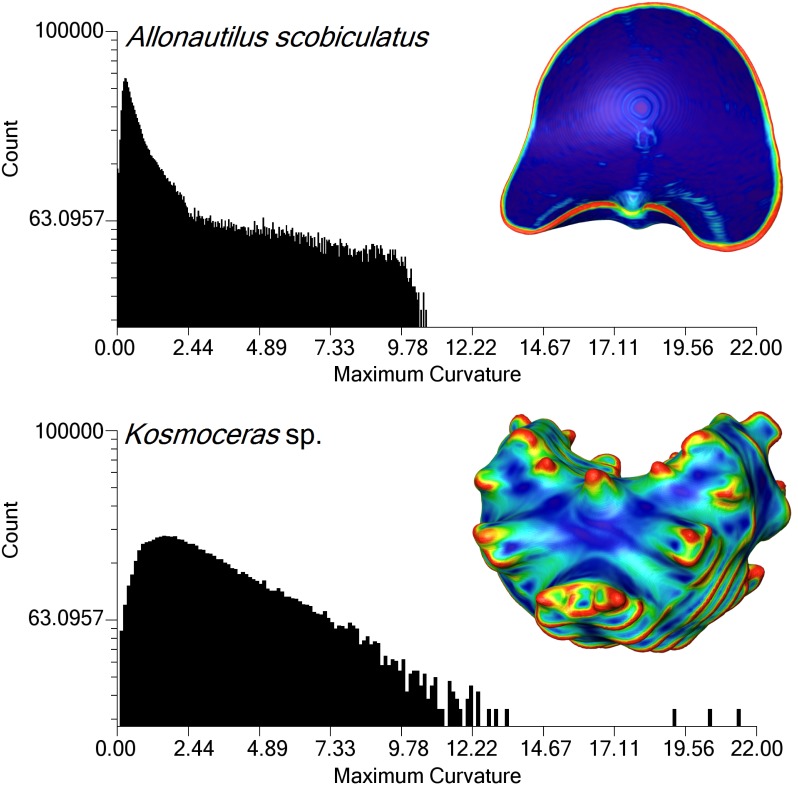
Comparison of the curvature between one chamber of *A*. *scrobiculatus* and *Kosmoceras* sp. Both chambers have similar volume and the chamber of *Kosmoceras* sp. was resampled to the same voxel size to make the datasets comparable. Curvature is measured at the vertices of the surface mesh. Overall, *Kosmoceras* sp. shows a consistently higher curvature over a greater percentage of its available surface area. Both chambers show highest curvature along the suture line.

The curvature across the face of the folded septa of *Kosmoceras* sp. is greater than an equivalently sized chamber of *A*. *scrobiculatus* (Fig 6). It can be seen that the suture line traces an area of highest curvature in both *Kosmoceras* sp. and *A*. *scrobiculatus* (Fig 6); extreme frilling of the septal margin may increase the relative length of this area of high curvature. Contrary to [32], *Kosmoceras* sp. does not show dramatically higher curvature than *A*. *scrobiculatus*; however, the consistently higher curvature over a larger percentage of the chamber surface and a potentially longer relative suture line could contribute to a quicker chamber reflooding system [32].

### Evolution of high SA_C_:V_C_

We anticipated that the Mesozoic ammonites were going to possess the highest SA_C_:V_C_, however, the Paleozoic goniatite *Arnsbergites* sp. shows similar values compared with the Jurassic ammonites (Fig 2B). Above three millimeters the SA_C_:V_C_ of ammonoids seems to converge with the chamber ratios seen in *S*. *spirula*, which themselves would converge with the value of *A*. *scrobiculatus* at an estimated shell size of 20–30 mm (Fig 2B). This convergence is an unexpected trend [26] as *S*. *spirula* has the simplest septal morphology: a semi-hemispherical dome-shaped structure, compared to the multi-lobed septa of *Kosmoceras* sp. (Fig 1A and 1H). *S*. *spirula* also possesses a nearly circular whorl cross-section throughout most of its ontogeny as well as a whorl interspace and smooth inner conch surface whereas *Kosmoceras* sp. shows strong shell ornamentation that affects the inner conch surface and constant whorl overlap, which is expected to increase the relative surface area of the chambers [25,48]. At sizes above 3 mm, ammonoids do not seem to possess a higher functional surface area (Fig 2B), or indeed higher absolute surface area (Fig 2D), in the phragmocone chambers.

Although ammonoids seem to have a relatively “normal” SA_C_:V_C_ compared to *S*. *spirula* in later ontogeny, they possess a high SA_C_:V_C_ in earliest ontogeny. What then, if anything, does this seemingly characteristic high SA_C_:V_C_ in ammonoids reflect? A suite of morphological changes during the origination of ammonoids from their bactritid ancestors, as well as the early evolution of ammonoids towards tightly coiled planispiral shells, has been connected to the evolution of a rapid, high fecundity reproductive style similar to that of modern coleoids [38,55,56]. In contrast, the reproductive strategy of nautilids is characterized by slow growth, multiple reproductive events over the life of the animal, and relatively large hatchlings which closely resemble the adult animal [36,56,57]. The high SA_C_:V_C_ shown in the studied ammonoids may be another expression of a general evolutionary trend towards a high fecundity reproductive strategy as high SA_C_:V_C_ would increase potential fluid exchange rates that could permit quicker growth.

Ward *et al*. [58] found that chamber formation in *Nautilus macromphalus* occurs when the previous chamber is nearly half emptied of cameral fluid, the coupled-decoupled transition point. This observation allowed a very precise prediction on the timing of new chamber formation; although, it was observed that new chambers can be formed under certain conditions without the previous chamber being emptied at all [36]. It stands to reason, however, that because the shell’s primary function is a buoyancy device, if the animal continued to grow without emptying the chamber, then it would accrue too much weight to remain buoyant. Speed of chamber emptying is therefore one necessary factor limiting potential growth rate. The relatively high SA_C_:V_C_ and SA_S_:V_C_ seen in ammonoids therefore fits well with an evolutionary trend towards rapid early growth as both would increase the potential speed of chamber emptying.

Growth rates in some coleoids are known to be very sensitive to water temperature [59,60]; however, juveniles tend to show a consistently higher, exponential, growth rate which decreases in later ontogenetic stages [61–65]. Squids invest more energy in growth than do other iteroparous mollusks resulting in relatively higher overall growth rates through life [66]. As ammonoids develop a more coleoid-like reproductive strategy [38] we might expect changes towards a more coleoid-like growth pattern.

If this idea is true we would expect to see a trend towards increasing SA_C_:V_C_ and SA_S_:V_C_ between the bactritids and ammonoids and during the evolution of the relatively tightly coiled, planispiral shell of early ammonoids. As noted before, the high SA_C_:V_C_ and SA_S_:V_C_ seen in the early ontogeny of the ammonoid shell decreases through ontogeny, eventually equaling that of *S*. *spirula* and, presumably, given a large enough specimen, *A*. *scrobiculatus* (Fig 4A). This means that the potentially rapid emptying rates seen in early shell chambers decreases significantly when the animal grows, losing this function when the animal enters later ontogenetic stages. This fits with the general decrease in growth rates of coleoids through ontogeny [36,63]. It must be noted that rapid early growth does not demand that the animal had a shorter total life-span or even that the high growth rate was selected for in order to reach sexual maturity faster, since the high functional surface area operates only in small sizes and disappears before the animal actually reaches maturity.

Rapid early growth could also be a protective mechanism, evolved to compensate for decreasing hatchling size [38] and to protect from predators. [67,68] hypothesized that the evolution of rapid early growth in coleoids, along with their particular reproductive strategy, was a response to predation, as larger animals are less likely to be eaten and animals are more vulnerable to predation in early ontogeny [69,70]. The hatchling size of nautilids is an order of magnitude greater than the hatchling size of ammonoids and *S*. *spirula*. The hatchling size of *N*. *pompilius* is between 2–3 cm [71] while the hatchling size of our *Arnsbergites* sp. is 0.71 mm, *Amauroceras* sp. is 1.09 mm, *Cadoceras* sp. is 0.9 mm, and *Kosmoceras* sp. is 0.86 mm. This small size confines ammonoid hatchlings to low Reynolds numbers where jet propulsion is known to be much less effective relative to the adult because of the low relevance of inertial effects at these low Reynolds numbers [41,72,73]. Smaller forms also have higher energetic costs for locomotion [74] and decreased metabolic efficiency compared to larger forms [75]. This suggests a selective advantage towards rapid early growth when the hatchling size is small. *S*. *spirula* would also hatch with a small shell, diameter of approximately 1.5 mm for our *S*. *spirula*, however, as this is an internal shell it does not necessarily directly reflect hatchling size.

To test if ammonoids possessed a persistently high growth rate, studies focusing on the ontogenetic change in the SA_S_:V_C_ are necessary, as SA_S_:V_C_ in this study was confined to a single ammonite specimen, which was unfortunately not the largest ammonite studied, due to preservation.

### Septal Complexity

The evolution of septa complexity is, perhaps, the most debated trend in ammonoid evolution, encompassing decades of research; ideas of the function of complex septal are summarized in [20]. While we do not directly address septal complexity in this paper, septal complexity is one of three morphological features that control chamber volume and surface area. The other parameters, shell wall shape and septal spacing, show a complex relationship to both SA:V and Vogel Number (S1 Fig) and we propose septal complexity contributes a non-negligible amount to both of these values though we cannot say to what degree it contributes. However, as chambers increase rapidly in volume, and as the septa only border the chamber on two sides, the influence of septal complexity on chamber morphology should decrease through ontogeny. The increase in septal complexity does increase the curvature of the septal face, however (Fig 6), and may contribute to survivability by increasing the tolerance of the animal to shell loss [22,32].

If septal complexity is biologically constrained by the morphology of the shell [76,77], then the evolutionary drive to enhance early physiological function in the shell that we propose can, at least partially, explain the origin of septal complexity as a consequence of high SA_C_:V_C_/SA_S_:V_C_. A correlation between conch shape and septal complexity has been observed and can be interpreted in favor of this idea [38,78–80]. Covariation between morphological features, including septa, such as Buckman’s laws of covariation demonstrate the presence of developmental constraints in shell morphology [78,81,82].

We suggest that the initial evolution of folded septa from a hemispherical septum contributed to a chamber morphology that allowed faster growth of the hatchling, but we do not disregard other existing ideas of septal complexity. We postulate that conch morphology, septal complexity, and septal spacing would covary but any of these features can be shaped by variable selective pressure. Conch morphology may be shaped by hydrodynamics [83–85] or further folding of the septa, seen in later Mesozoic forms may be driven by mechanical strength [86] or enhancement of curvature (Fig 6). Evolutionary shaping of these parameters will induce changes in the other parameters that would not necessarily be functional but a consequence of growth. SA_C_:V_C_ may reflect this constrained growth. The increasing complexity of septa and increase in the shell ornamentation of *Kosmoceras* sp. does not cause any shifts in the SA_C_:V_C_ (Fig 4A). We expect all chambered shells to show stable trajectories through ontogeny, despite changes in conch or septal morphology, under normal growth (Fig 2). This is not a controversial statement as it merely says that we do not expect rapid changes in size and morphology at any single growth stage (chamber). The SA:V ratio is a quantitative way of phrasing this idea that can be tested. Furthermore, Vogel number can be used to test for changes in the chamber morphology through ontogeny, a constant Vogel number indicates no morphological change of the chamber.

Interestingly, comparisons between the two *S*. *spirula* specimens used in this study defy this trend. One specimen shows an abrupt shift in both volume and surface area (Fig 2C and 2D) and a sudden perturbation in the SA_C_:V_C_ that persists for about 5 chambers (Fig 4A). The shift and subsequent displacement is related to a sudden change in the morphology of one pathological chamber that has been crushed. While we suggest normal growth will always result in stable surface area, volume, and SA_C_:V_C_ trends due to size scaling and growth limits, sudden displacements in these trends may indicate the presence of pathologies—indicating either predation or sudden, massive environmental perturbations (e.g., temperature, salinity, food supply). The shifts in these trends in *Arnsbergites* sp. may be related to sudden changes in septal spacing and may be indicative of environmental stress. Kinks in these trends may indicate certain life events. The two kinks seen in *A*. *scrobiculatus* and *N*. *pompilius* may indicate the hatching moment (Fig 2A).

### Mechanical resistance and shell internalization

One of the most common explanations for the evolution of septal complexity is that the increase in septal folding increased the mechanical resistance of the shell against hydrostatic stress [19,86–89]. While addressing this idea is beyond the scope of this paper, it is interesting to note that the initial evolution of a complex septum from a simpler, dome-shaped septum decreases mechanical strength [21,32]. The evolutionary increase in septal complexity may be due to mechanical resistance, though a lack of correlation between habitat and septal complexity challenges this idea [23,90]. Increased shell and septal thickness also influence mechanical resistance to hydrostatic pressure; however, comparing *Kosmoceras* sp. and *S*. *spirula* shows that for equivalent diameters, *Kosmoceras* sp. has a thicker shell wall but a slightly thinner median septal thickness. An extension of the mechanical explanation is that the septa increase resistance to point forces such as those from bites [21,32]. This idea however has been challenged [90] and recent attempts to find a correlation between septal complexity and rate of survival from shell breakage—which would be expected if increased septal foldings protected from predation—have failed to find a significant link [91]. Mechanical hypotheses therefore are unlikely to explain the initial evolution of complexity; however, our comparisons between *S*. *spirula* and the derived ammonoids suggest that SA_C_:V_C_ and SA_S_:V_C_ enhancement may be an important factor in this event. Internal shells do not show the complex morphologies seen in either ammonoids or nautilids. If septal morphology is connected to hydrostatic pressure then cephalopods living in deep waters should have more complex septa than shallow water forms regardless of possessing an internal or external shell. If however, septal complexity is viewed as an aspect of growth, then this relationship need not exist. Indeed, *S*. *spirula* has the simplest septal morphology but can dive to a depth of ~1000 meters [92], a depth deeper than most depth estimates for ammonoids [8,93]. This view permits some speculation about the evolution of an internal shell.

The soft body of ammonoids and nautiloids is encased in the shell throughout ontogeny and therefore the size of the soft body is strongly limited by the size of the shell; therefore, growth of the animal is strongly connected to the growth of the shell as discussed before. The internalization of the shell may result in a partial decoupling of the growth of the shell and the growth of the animals soft body. The animal is no longer constrained in a rigid container, allowing an increase in soft body growth without necessarily growing the shell. The internal shell still functions as a buoyancy device; however, as the soft body is no longer within the shell, the evolution of a mantle-pump propulsion system can compensate for the increase in weight without a proportional increase in phragmocone volume. The mantle-pump system of coleoids involves the inflation of the soft body and allows greater total velocity and is more energetically efficient than the nautilid propulsion system [94–98].

## Conclusions

1. The chamber surface area to chamber volume ratio (SA_C_:V_C_) of ammonoid chambers shows an initially high value compared to *S*. *spirula* and nautilids; however, at sizes larger than about 3 mm, the ratio of ammonoids becomes nearly identical to the values of *S*. *spirula*. Larger ammonoid shells are expected to show ratios similar to nautilids at sizes around 20–30 mm.
2. The siphuncle surface area to chamber volume (SA_S_:V_C_) is higher in ammonoids than in either *S*. *spirula* or the nautilids. This confirms the increase in functional area of the siphuncle that can be explained by the migration of the siphuncle to the ventral edge of the chamber.

We propose that the initial high SA_C_:V_C_ and persistently high SA_S_:V_C_ in ammonoids reflects a trend towards increased growth rates in early, post-hatching stages. Ammonoids have been found to have evolved a more coleoid like reproductive strategy relative to extant nautilids [38,56]. Coleoids are known to have a higher growth rate in post-hatching juveniles which decreases through ontogeny [63]. High early growth rates in ammonoids may therefore be supplementary to a high fecundity reproductive strategy.

Septal complexity is one of three morphological characters that influence SA_C_:V_C_ and SA_S_:V_C_, the other two being septal spacing and conch morphology. Therefore, septal complexity may have contributed to the enhancement of SA_C_:V_C_ and SA_S_:V_C_ in the early shell. Scaling eliminates this benefit at moderate sizes; however, we further demonstrate that septal folding increases the curvature of the septal face and elongates the region of highest curvature, which is traced by the suture line.

The presented hypothesis is strengthened by the fact that a multi-lobed septum would be mechanically weaker compared to the ancestral, hemispherical dome-shaped septa [21,32]. We further suggest that septal morphology, conch morphology, and septal spacing are covarying parameters which are reflected by stable surface area, volume, and SA_C_:V_C_ trends through ontogeny. Displacements of this trend may indicate pathologies and stressed environments while single, point deviations in this trend may indicate life events such as hatching. The observation of high functional area being limited to the early shell also presents potential problems for some physiological explanations for septal function.

## Supporting Information

S1 Fig Comparison of various expansion rates against septal angle and septal angle expansion rate for the ammonite *Amauroceras* sp. The expansion rate of a parameter is defined as the value of that parameter in one chamber divided by the value of the same parameter in the preceding chamber; e.g. Vc expansion rate = Vn/Vn-1 where Vn is the volume of chamber n. Interestingly while changes in septal angle have the highest correlation with changes in volume (correlation coefficient of 0.60), the correlation with our functional parameter (S_C_/V_C_) and our shape parameter (Vogel number) is lower, -0.58 and -0.29 respectively.
(EPS)

S1 Table Volumetric Data for All Specimens. (ODS)
